# Hyperkalemia masking left ventricular hypertrophy in electrocardiogram in a patient with end-stage renal disease

**DOI:** 10.4103/0971-4065.41284

**Published:** 2008-01

**Authors:** V. V. Sai Naresh, V. Ramakrishna, P. Ramakrishna, V. Siva Kumar

**Affiliations:** Department of Nephrology, Sri Venkateswara Institute of Medical Sciences (SVIMS), Tirupati - 517 507, Andhra Pradesh, India

**Keywords:** Electrocardiography, end stage renal disease, hyperkalemia, left ventricular enlargement

## Abstract

A patient of severe chronic renal failure presented with hyperkalemic electrocardiographic (ECG) changes and hyperkalemia. Following a session of hemodialysis, when he reverted to normokalemia, the repeat ECG revealed left ventricular hypertrophy (LVH). Thus we confronted with a situation of hyperkalemia, masking the LVH on ECG initially and when the hyperkalemia was corrected with dialysis the LVH showed in ECG. The plausible explanation for this electrophysiological behaviour was offered.

The electrocardiographic (ECG) changes suggesting left ventricular hypertrophy and hyperkalemia have been well established. However, there appears a paucity of understanding regarding the behavior of ECG in a compounding situation of hyperkalemia in the background of left ventricular hypertrophy. Keeping in view a similar situation encountered by us in a patient, we propose to submit this observation with a plausible scientific reasoning.

## Case Report

**Fig. 1:**
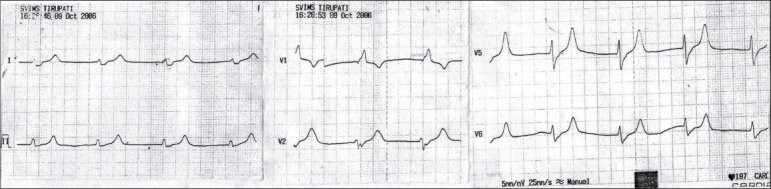
Showing features suggestive of hyperkalemia (S.K 7 meq/I): Rate 63/min, absent P waves, junctional rhythm, with tall T waves

**Fig. 2:**
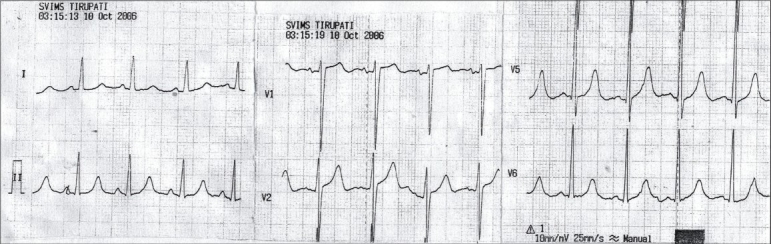
Showing features of left ventricular hypertrophy: Nornal sinus rhythm, rate 73/min. left axis deviation, reappearance of P waves, high amplitude QRS complexes, S in V1 30 mm, R in V6 23 mm suggesting left ventricular hypertrophy and decrease in T wave amplitude. This ECG was taken after hemodialysis (S.K 5.3 meq/l)

A 55-year-old male presented to the emergency room with pulmonary edema. He did not provide the history of any comorbid illnesses and was not on any medication. On examination, he was found to have anemia orthopnea, cyanosis and anasarca. The patient exhibited bradycardia and hypotension. His ECG on admission revealed a heart rate of 63/min, absence of P waves, junctional rhythm and tall T waves [Fig. 1]. His hemoglobin was 6.7 g/dl; total count, 14,200/m^3^; serum creatinine, 9.9 mg/dl; blood urea, 206 mg/dl; serum sodium, 139 meq/l and serum potassium, 7 meq/l. Appropriate antihyperkalemic measures and hemodialysis was initiated. Following are the biochemistry of posthemodialysis serum: serum creatinine, 5.6 mg/dl; sodium, 136 meq/l; potassium, 5.3 meq/l and calcium, 8.9 mg/dl. A repeat ECG performed in the patient after hemodialysis revealed normal sinus rhythm, heart rate of 73/min, left axis deviation, reappearance of P waves, high-amplitude QRS complexes suggestive of left ventricular hypertrophy and a decrease in the T wave amplitude [Fig. 2]. Ultrasound of the abdomen revealed the size of the right kidney, 10.1 × 3.8 cm and that of the left kidney, 7.8 × 3.5 cm; echogenicity increased and maintained corticomedullary differentiation in the right kidney; the left kidney contracted with loss of corticomedullary differentiation and no hydronephrosis. Two-dimensional echocardiography revealed concentric left ventricular hypertrophy. The patient improved symptomatically after three sessions of hemodialysis and was discharged on request while in a stable condition.

## Discussion

Physiologically, the electrical activation of the ventricles may be depicted in a simplified presentation as a small initial vector from the left to right through an interventricular septum, followed by a large vector from the left to the right through the free wall of the ventricle. Under condition of left ventricular hypertrophy, the abnormalities of the QRS complex noted were increased magnitude of QRS deflection and an increase in the left ventricular activation time. The increased magnitude of the left ventricular activation time was attributed to factors such as increased left ventricular muscle mass, increased left ventricular muscle surface and proximity of the left ventricle to the chest wall.^1^,^2^

In hyperkalemia, a reduction occurs in the cardiac resting membrane potential due to decrease in the transmembrane potassium gradient, thereby inactivating sodium channels and decreasing the conduction velocity. This leads to inexcitability of the cardiac muscle fiber, resulting in broadening of QRS, with a decrease in the amplitude of P and QRS complexes. Eventually, sine wave and cardiac arrest in diastole occur.^3^–^5^

In a compounding situation of hyperkalemia, manifesting in an already existing left ventricular hypertrophy, a possible explanation may be that when the conduction velocity and the excitability of the cardiac muscle decreases in hyperkalemia, the manifestation of increased amplitude of the QRS complex suggestive of left ventricular hypertrophy is masked. However, once hyperkalemia reverts to normokalemia, the hypertrophied left ventricle manifests its changes in the ECG.

This case report highlights the interaction of two opposing coexisting situations such as hyperkalemia on the background of left ventricular hypertrophy, which can manifest initially with hyperkalemic changes in the ECG and after correcting to normokalemia via dialysis, the left ventricular hypertrophy may reappear in the ECG.
